# HIV and the growing health burden from noncommunicable diseases in Botswana: modelling study

**DOI:** 10.7189/jogh.09.010428

**Published:** 2019-06

**Authors:** Markus Haacker, Till Bärnighausen, Rifat Atun

**Affiliations:** 1 Department of Global Health and Population, Harvard T.H. Chan School of Public Health, Harvard University, Boston, Massachusetts, USA; 2 Centre for Global Health Economics, University College, London, UK; 3 Heidelberg Institute of Public Health, University of Heidelberg, Heidelberg, Germany; 4 Africa Health Research Institute, KwaZulu-Natal, South Africa; 5 Department of Global Health and Social Medicine, Harvard Medical School, Harvard University, Boston, Massachusetts, USA

## Abstract

**Background:**

The “greying of AIDS” – the aging of the population living with HIV who benefit from antiretroviral treatment (ART) and the emergence of age-related non-communicable diseases (NCDs) – has been well documented. The emerging health systems challenges – eg, the implications of HIV on the disease burden from NCDs on the population level, and the evolving role of HIV as a co-morbidity or co-existing disease of various NCDs – are less well understood. The paper elucidates these challenges by providing a quantitative analysis of HIV-NCD interactions for Botswana.

**Methods:**

We projected the prevalence of HIV and of selected NCDs in Botswana using demographic and HIV-specific estimates building on data on the state and the dynamics of the HIV epidemic, using the Spectrum modelling software, and extrapolating on estimates of the prevalence of NCDs from the 2015 global burden of disease (GBD).

**Results:**

HIV has slowed down overall population aging and thus has attenuated the growing burden of many NCDs so far, because cohorts reaching old age have been decimated by AIDS-related mortality in the 1990s and early 2000s. Aging and the rise in the prevalence of NCDs, however, will accelerate rapidly from about 2030 because of reduced attrition of cohorts living with HIV since the start of the ART scale-up in Botswana. While HIV prevalence will decline over time, the health needs of people living with HIV will become more complex. HIV prevalence among the growing populations affected by various important NCDs will not decline for decades, because of the aging of the population living with HIV and interactions between HIV, ART and NCDs.

**Conclusions:**

Even though HIV prevalence is projected to decline steeply to 2030 because of reduced HIV incidence, the prevalence of HIV among people affected by many of the most important NCDs will increase or barely change. While the health care needs of people living with HIV will increase and become more complex, HIV will also emerge as a key factor complicating the management of the growing burden of NCDs. Health systems will need to prepare for the challenge of large numbers of patients living with both HIV and NCDs.

The “greying of AIDS” – the increasing age of the population living with HIV owing to improved survival of people living with HIV (PLWH) who have benefited from antiretroviral treatment (ART), and reduced incidence of HIV – has been well documented [1-4]. As a consequence of this phenomenon, the health needs of the population living with HIV are changing as ageing PLWH increasingly develop noncommunicable diseases (NCDs) like cancers, cardiovascular diseases, digestive diseases, and diabetes mellitus [5-8].

The presence of NCDs complicates HIV-specific care and treatment, while the presence of HIV is a complicating factor in the management of NCDs [9,10]. Moreover, HIV and antiretroviral therapy (ART) may both contribute to development of some NCDs and the occurrence of HIV-NCD comorbidities [11]. In many countries, these changes occur against the backdrop of rapid aging of the general population, and consequently an increasing burden from NCDs across the total population [1,12-17].

The paper addresses the health systems challenges posed by the intersecting trends of the HIV epidemic and major NCDs. To this end, it goes beyond the literature on the “greying of AIDS” in two directions. First, we look at the intersections of HIV and NCDs symmetrically, addressing the increasing burden of NCDs among PLWH, but also the evolving role of HIV among the populations affected by various NCDs. Second, we take into consideration demographic effects of HIV. Population cohorts reaching old age now have been decimated by past AIDS-related mortality, a factor that is slowing down increases in the prevalence of age-related NCDs. However, this effect is reversed as cohorts reaching old age increasingly benefit from the availability of treatment.

We investigate the health systems challenges posed by the “greying of AIDS” and the increasing HIV-NCD comorbidities in Botswana. We selected Botswana for our analysis because we expect these challenges to be particularly important in this country. Botswana has a very high HIV prevalence, has experienced very high AIDS-related mortality in the past, and has been one of the most successful countries in terms of providing access to treatment for PLWH [18]. Moreover, because underlying (ie, excluding the impacts of HIV) life expectancy is among the highest in sub-Saharan Africa, and given the early and comprehensive scaling-up of HIV treatment, we expect aging and comorbidity development to be especially pronounced in Botswana’s HIV-positive population.

Both HIV and the growing burden of NCDs, and their intersections, have been recognized as key health policy challenges in Botswana. HIV and NCDs are highlighted as the two key health challenges in the national development strategy [19], and – while HIV has been a dominant issue over the last decades – the government has also formulated a comprehensive NCD strategy [20]. Moreover, the challenges of addressing HIV and NCDs are increasingly seen as interlinked, as demonstrated by the pointers to NCDS in the HIV/AIDS strategy [21] and the recent decision to assign responsibilities on “prevention and health promotion aspects of NCDs” to the National AIDS Coordinating Agency [22].

Because we are interested in the health systems challenges posed by HIV and NCDs – and because both HIV and most NCDs are chronic diseases –, our focus is on the prevalence of, and inter-relationships between, these diseases rather than on mortality. The quantitative analysis blends epidemiological and demographic work files based on “Spectrum” software underlying official estimates of the state of HIV/AIDS in Botswana, and estimates of the prevalence of NCDs from the Global Burden of Disease (GBD) 2015 database [18,23-25].

Results are presented in two steps. First, we discuss the changing age composition of the population living with HIV and the demographic implications of past and projected AIDS-related mortality. Second, we explore the implications of the ageing of AIDS in terms of the increasing role of NCDs among HIV-affected individuals, and the role of HIV among the populations affected by NCDs, across the full spectrum of NCDs covered in the GBD 2015 estimates. We also discuss the role of HIV as a contributor to the prevalence of selected NCDs (diabetes, ischemic heart disease).

## METHODS

The analysis consists of two building blocks. First, demographic and HIV-specific estimates and projections were generated using Spectrum software, building on the file underlying the latest estimates on the HIV epidemic published by UNAIDS. Second, projections on the prevalence of NCDs were obtained by extrapolation of age-specific estimates from the GBD 2017 database [24,25]. These two building blocks are then combined to obtain estimates of the prevalence of NCDs among people living with HIV, and of the prevalence of HIV among people with NCDs.

Estimates and projections of the state of the HIV epidemic were generated using the “AIDS Impact Module” of the Spectrum software package [23]. This software is used by UNAIDS and national authorities to derive comprehensive estimates of the history and state of the HIV epidemic from available data, and – conditional on assumptions on treatment access and the path of HIV incidence – can also be used to generate projections. The “AIDS Impact Module” is a compartmental model of the HIV epidemic, in which PLWH progress by age and stage of disease, with age- and sex-dependent transition rates. It is embedded in a demographic framework which pulls relevant data from the UN World Population Profile [26].

Specifically, we build on the estimates by the national authorities and UNAIDS, which form part of the global estimates published by UNAIDS in 2018 [18,27]. The Spectrum work file which had been used to generate these estimates was obtained and is publicly available from UNAIDS (http://apps.unaids.org/spectrum/). Our analysis utilizes Spectrum 5.63, ie, the version which has been used for the UNAIDS 2018 estimates.

The UNAIDS Spectrum work file contains validated estimates through 2017, but the projections (through 2025) contained therein are based on simple extrapolation of most policy variables, and are not (and not meant to be) based on a meaningful forward-looking policy analysis. To adapt the file to our purposes, we extended the time horizon from 2025 to 2050 (by changing the final year in the Spectrum “projection manager” module) [28], and aligned projections on key policy variables with the “National Multisectoral HIV and AIDS Response Strategic Framework 2018 – 2023” (NSF). Specifically, we assume that treatment coverage increases to 95% of adults (ages 15+) living with HIV (91% for men, 98% for women) by 2023, from 84.5% in 2017 (72.4% for men, 93.5% for women), and remains constant from 2023. The NSF does not provide a concrete target for treatment coverage (other than *exceeding* a benchmark of 90%), but the declines in AIDS-related deaths targeted in the NSF suggest ongoing improvements in treatment access. The assumed increase to 95% in our paper is our interpretation of these policies.

For HIV incidence, we adopted the targets from the NSF through 2023 (declining from 1.1% of adults at ages 15+ in 2017 to 0.2% by 2023, driven by the ongoing expansion of treatment and high rates of viral suppression among those receiving treatment [29]. From 2023, we assumed that HIV incidence declines gradually in proportion with HIV prevalence. The assumptions on HIV were inserted into the Spectrum file using the “direct incidence input” option. Because incidence affects prevalence (and – according to our assumptions – feeds back into incidence), this progress was repeated for several iterations until the projections on prevalence (and thus assumed incidence) converged.

The choice of Spectrum for generating the demographic projections deserves a comment. While there are alternative sources of demographic projections, notably the UN World Population Profile (WPP), Spectrum actually imports its demographic assumptions from a “no AIDS” version of the World Population Profile, while the WPP includes an HIV/AIDS module that is similar to Spectrum. We used Spectrum for the demographic projections to ensure they reflect the latest estimates on the state of the HIV epidemic and assumptions on the policy outlook, and to obtain clear-cut estimates of the impact of HIV.

Age- and sex-specific estimates on the prevalence of NCDs through 2017 were obtained from the GBD 2017 database, a global collaboration to provide estimates of fatal and non-fatal health losses across countries, by sex, age, and – in its latest iteration – 354 diseases and injuries [24,25]. Especially for smaller countries like Botswana, it is by far the most comprehensive source on the prevalence of health conditions. This is achieved by meta regressions over a large body of evidence – from population surveys, medical and insurance databases, and disease-specific academic studies and reviews [25]. As a consequence, GBD estimates – akin to the outcomes of a cross-country regression – may differ from specific observations for that country.

For this reason, we have also reviewed country-specific data which could inform our analysis. The most important alternate data source on NCDs in Botswana is the 2014 STEPS survey which however focuses on the prevalence of NCD risk factors (many of which are self-reported) rather than prevalence of NCDs per se [30]. For prevalence of diabetes, however, this survey reports estimates which are less than one-half of the estimates contained in the GBD 2017 database. We have adopted the estimates of prevalence of diabetes from the 2014 STEPS survey, as the most authoritative nationally validated data (see Appendix S1 in **Online Supplementary Document**, especially Figure SA1). These national estimates are also consistent with the other international database on the prevalence of diabetes, the IDF Diabetes Atlas [31]. Because diabetes is a major risk factor for kidney disease, we also adjusted estimates on chronic kidney disease for lower prevalence of diabetes (see Appendix S1 in **Online Supplementary Document**, especially Figure SA2).

We included all “level 2” categories of NCDs covered in the GBD estimates (**Table 1**); for selected NCDs (especially where there is some evidence on links between long-term survival with HIV and the prevalence of these diseases) we included further sub-categories. There are several approaches which could be used to project prevalence of NCDs. Most results reported in this paper assume that age-specific prevalence remains constant from 2017. An alternative would be to extrapolate prevalence levels based on recent trends in prevalence. We followed the former approach, because age-specific prevalence generally changed slowly, and the differences in projections based on the alternative approaches are therefore small. As age-specific prevalence rates underlying the projections are assumed constant, our projected changes in the prevalence of NCDs can then be clearly attributed to changes in the age composition of the relevant populations. A comparison with findings for an alternative assumption of extrapolating through 2040 based on trends in age-specific prevalence of NCDs in 2010-2017 (Tables S2 and S3 in **Online Supplementary Document**) shows that the two approaches return similar estimates, and none of the essential findings of the paper would need to be changed if this approach were adopted.

**Table 1:** Prevalence of selected NCDs among people living with HIV, 2015-2040*

	Prevalence (%)						
	**2015**	**2020**	**2025**	**2030**	**2035**	**2040**	**Relative increase, 2015-2040 (%)**
Neoplasms/Cancers	0.6	0.7	0.9	1.1	1.2	1.4	152.4
Cardiovascular diseases:	5.5	6.7	8.4	10.4	12.8	15.5	180.8
-Ischemic heart disease	1.3	1.7	2.2	2.8	3.5	4.2	216.4
-Cerebrovascular disease	0.9	1.1	1.5	1.9	2.4	2.9	234.1
-Hypertensive heart disease	0.1	0.1	0.1	0.1	0.2	0.2	285.2
-Cardiomyopathy and myocarditis	0.1	0.1	0.2	0.3	0.4	0.5	354.8
Chronic respiratory diseases	7.9	9.1	10.7	12.6	14.5	16.5	108.7
Digestive diseases:	34.6	36.4	37.9	39.2	40.2	41.2	19.0
-Cirrhosis and other chronic liver diseases	23.6	25.1	26.2	27.2	28.0	28.7	21.3
Neurological disorders	48.8	48.6	48.0	46.8	45.4	44.2	-9.5
Mental disorders	14.4	14.5	14.6	14.6	14.6	14.6	1.4
Substance use disorders	3.2	3.1	2.9	2.7	2.5	2.3	-27.8
Diabetes and kidney disease:	13.7	15.7	18.5	21.4	24.5	27.7	101.9
-Diabetes mellitus	3.2	3.8	4.7	5.6	6.6	7.5	134.0
-Chronic kidney disease	11.9	13.5	15.7	18.1	20.7	23.5	97.4
Skin and subcutaneous diseases	24.7	25.5	26.8	28.5	30.7	33.3	35.1
Sense organ diseases	38.3	42.3	47.5	52.6	57.4	61.9	61.6
Musculoskeletal disorders	17.6	19.8	22.7	25.3	27.8	30.2	71.7
Other non-communicable diseases	69.0	69.5	69.9	70.1	70.3	70.4	2.0

NCD – non-communicable disease

*Table shows all “level 2” NCD categories from GBD 2017, and a selection of more specific diseases (indented). The relative increase is calculated as the ratio of prevalence in 2040 and 2015, respectively, minus one, multiplied by 100.

We report estimates and projections on the prevalence of NCDs among PLWH (and among the HIV-negative population), defined as average across sexes and age brackets, weighted by the size of the respective populations. Except for the discussion on direct linkages between HIV status and prevalence of NCDs (see below and Annex), we assume that HIV status and NCD prevalence are uncorrelated (controlling for sex and age), so that the prevalence of NCDs is the same for PLWH and among the HIV-negative population within age/sex brackets. The prevalence of HIV among people affected by NCDs is calculated as average across the population affected by NCDs, weighted by the size of sex and age brackets, again assuming that HIV status and NCD prevalence are uncorrelated (controlling for sex and age).

Where there is reason to expect that age-specific prevalence rates will depart from observed levels or trends, it would make sense to accommodate such changes in the projections. For example, policies towards reaching the 25×25 targets (of achieving a 25% reduction in premature mortality from NCDs by 2025) might result in such changes [32]. We have not attempted such projections. The 25×25 framework involves a mix of measures to reduce incidence of NCDS and to improve survival with NCDs, and cannot easily be mapped into assumptions on prevalence, and modelling efforts inspired by 25×25 targets focus on mortality rather than prevalence of NCDs [33]. Similarly, the national NCD strategy describes strategies to mitigate NCD risks and to improve health outcomes among people affected by NCDs, but includes a “no increase” target for prevalence of diabetes and obesity [20].

We do however address the potential implications of direct linkages between HIV and NCDs. If HIV or exposure to ART results in an increased risk of developing some NCDs, then this NCD will be a more common co-morbidity among PLWH, and HIV will be a more common co-morbidity among people affected by this NCD than among people not affected by it. We therefore provide alternate estimates, introducing a multiplier that differentiates prevalence of selected NCDs among PLWH and people not living with HIV, controlling for sex and age group, for ischemic heart disease and diabetes mellitus where empirical evidence is available, including studies from outside high-income settings (see Appendix S3 in **Online Supplementary Document**) [34-42]. For the projections, we do not distinguish PLWH on ART and without treatment, in light of the very high treatment coverage rate achieved and targeted in Botswana, especially for PLWH reaching old age. For this reason, we also do not account for the effect of HIV or ART on the body mass index (a risk factor for some NCDs). As treatment results in recovery of weight losses [43], and most PLWH in our projections will have been on treatment for a number of years (especially those at older ages when NCDs become more common), differences in body mass index (BMI) between HIV-positive and HIV-negative people will arguably not be a significant factor in the evolving burden of NCDs in Botswana.

We did not include a coefficient to account for the interactions of HIV and cancers in the calculations because – unlike for AIDS-defining cancers – the empirical evidence is diffuse and not clear-cut with regard PLWH who are receiving ART [44,45].

## RESULTS

### Population size and composition

The evolving age distribution of the population now reflects demographic and health changes over the last decades, including steep declines in mortality (especially infant and child mortality) between 1950 and 1980, a fertility transition which set in around 1980, and of course the impact of HIV/AIDS.

Child mortality declined from 20.9% in 1950 to 15.8% in 1965, and 9 .4% in 1980 [26]. Life expectancy at age 15 increased from 46 years in the early 1950s to 53 years around 1985 [26]. Additionally, the fertility transition set in around 1980 (preceding, but then exacerbated by the impacts of HIV/AIDS), with crude birth rates declining from 4.4% in 1980 to 2.8% in 2000 [26]. These factors contribute to high growth of the older population at present and over the next decades, either absolutely (owing to declining mortality) or relatively (declining fertility means that the weight of younger cohorts in the population distribution declines).

These changes, of course, are modified by the impact of HIV. AIDS-related mortality increased steadily in the 1990s, and peaked at about 5% of adults living with HIV (1.2% of total population at ages 15+) in 2000-2004, but has declined steeply (to 0.9% of adults living with HIV by 2015) since then as a consequence of the roll-out of treatment [27]. Nevertheless, cohorts reaching old age now are heavily depleted by HIV/AIDS. For example, past AIDS-related mortality has diminished the size of the population at ages 55 to 64 by an accumulated 20% as of 2015 [27].

**Figure 1:**
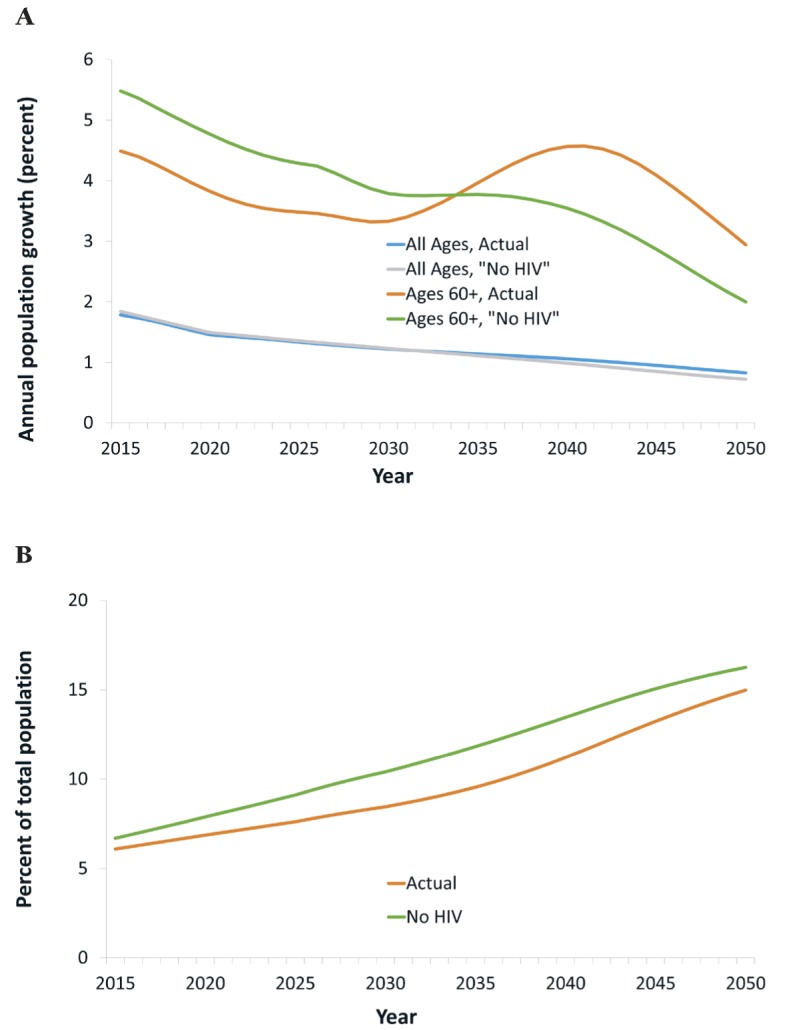
HIV and the size of the population at ages 60 and older. Panel A. Annual population growth. Panel B. Population at ages 60+.

In our projections, the total (ie, HIV+ and -negative) population at ages 60+ grows rapidly, at an average rate of 4% annually in 2015-2030 (**Figure 1**, panel A). HIV/AIDS mitigates this rapid growth, by up to 0.8 percentage points and an average of 0.6 percentage points annually in 2015-2030. Consequently, the share of the population at ages 60+ grows more slowly than it would without HIV/AIDS, from about 6.5% in 2015 to 9.0% by 2030 instead of 10.4% (**Figure 1**, panel B). This effect, though, will be reversed from about 2030, because cohorts reaching age 60 then are much less decimated by HIV/AIDS than older cohorts, owing to the widespread availability of treatment from around 2003. As a result, the growth rate of the population at ages 60+ reaches an all-time high, at about 5%, in 2040, of which one percentage point reflects the impacts of HIV/AIDS, especially the reversal in mortality among PLWH in preceding decades. In summary, HIV/AIDS is and has been mitigating the growth of the older population (and consequently increases in the prevalence of age-related NCDs), but it will become an accelerating factor in the future.

**Figure 2:**
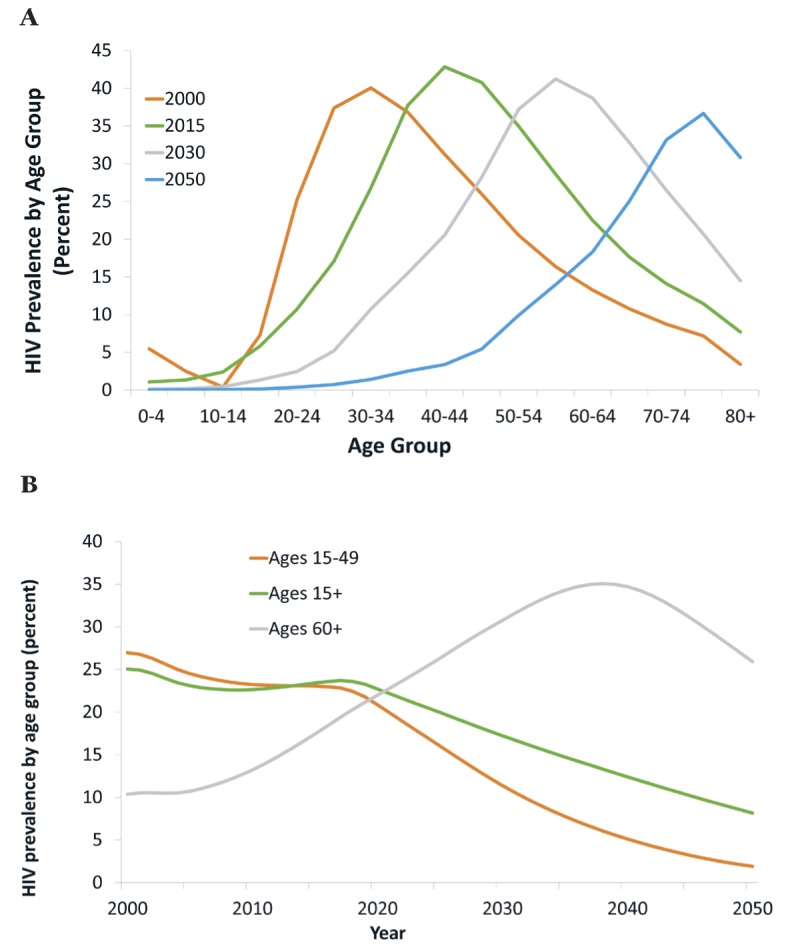
HIV prevalence across age groups. Panel A. HIV prevalence by age group, various years. Panel B. HIV prevalence by broad age group.

Beneath these changes in the total population, there are large changes in the composition of the population living with HIV. The age distribution of the population living with HIV is rapidly shifting to older age brackets (**Figure 2**, panel A). This is a consequence both of reduced mortality (implying little attrition, until old age, as cohorts of PLWH progress to higher age brackets), and lower HIV incidence (resulting in declining HIV prevalence among the young).

One aspect of this shift in the age distribution of PLWH is strong divergence in trends in HIV prevalence across age groups (**Figure 2**, panel B). While “headline” HIV prevalence (the number most frequently reported, relating to ages 15-49) declines steeply, eg, from 23% in 2017 to 11% in 2030, HIV prevalence for the adult population overall (ages 15+) declines much less – from 24% in 2017 to 17% in 2030. Meanwhile, HIV prevalence at ages 60+ increases steeply from 19% in 2017 to 31% in 2030, at which time it will be nearly three times higher than “headline” HIV prevalence, and increases to 35% by 2040 (7 times “headline” HIV prevalence).

**Figure 3:**
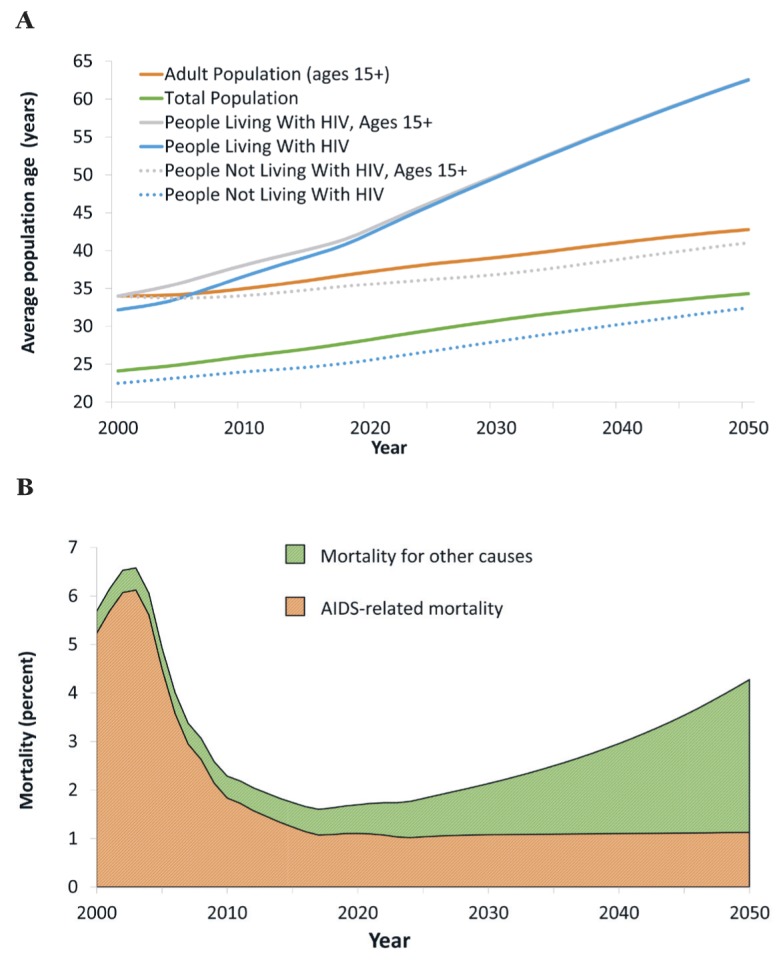
Average age and mortality. Panel A. Average population age. Panel B. Mortality among people living with HIV/AIDS.

While the population of Botswana ages overall (with or without HIV), the population living with HIV ages much faster than the total population. From 2017 to 2030, the average age of an adult increases from 37 years to 41 years (**Figure 3**, panel A). Over the same period, the average age of a person living with HIV increases from 40 years to 50 years. The ageing of the population living with HIV has consequences for projected mortality. Since 2005, mortality among PLWH has been driven by the scaling-up of ART, but in light of high treatment coverage rates already achieved this will no longer be a major driver of improvements in mortality. Instead, changes in mortality will be driven by the aging of the population living with HIV and non-AIDS-related deaths, primarily from age-related NCDs (**Figure 3**, panel B).

### Intersection of HIV and NCDs

Owing to the rapid aging of the population living with HIV, the prevalence of most NCDs among this population increases sharply over the coming years (**Table 1**, see Tables S1A and S1B in **Online Supplementary Document** for results by sex). For example, between 2015 and 2040, the prevalence of cancers, cardiovascular diseases, and diabetes mellitus increase by a factor of about 2.5, 2.8 and 2.3, respectively. These increases occur fairly steadily over this long period – meeting the increasing need for (non-HIV/AIDS) care of PLWH will be a recurring challenge. These findings are robust with regard to the assumption of a constant age-specific prevalence of NCDs. For example, if prevalence is extrapolated based on trends (2005-2015), the prevalences of cancers and of cardiovascular diseases increase by a factor of about 3.0 and 2.9, respectively (Table S2 in **Online Supplementary Document**).

The sharp increases in the prevalence of age-related NCDs among the population living with HIV need to be seen in the context of underlying demographic change, and declines in mortality for reasons not related to HIV/AIDS (see estimates and projections on average age of people not living with HIV, **Figure 3**, panel A). For this reason, the prevalence of most NCDs increases across the population, and not only for PLWH. To interpret the growing burden from NCDs among PLWH, it is therefore useful to compare it with projections on the population not living with HIV, and – because most PLWH are adults – with the adult population not living with HIV (**Table 2**).

**Table 2:** Prevalence of selected NCDs, adults (ages 15+), excluding people living with HIV, 2015-2040*

	Prevalence (%)						
	**2015**	**2020**	**2025**	**2030**	**2035**	**2040**	**Relative increase, 2015-2040 (%)**
Neoplasms/Cancers	0.5	0.5	0.5	0.5	0.5	0.6	29.6
Cardiovascular diseases:	5.5	5.7	5.8	6.0	6.3	6.7	23.0
-Ischemic heart disease	1.3	1.4	1.4	1.5	1.6	1.7	32.7
-Cerebrovascular disease	0.9	0.9	0.9	1.0	1.0	1.1	30.3
-Hypertensive heart disease	0.1	0.1	0.1	0.1	0.1	0.1	25.0
-Cardiomyopathy and myocarditis	0.1	0.1	0.1	0.1	0.2	0.2	30.3
Chronic respiratory diseases	7.3	7.4	7.6	7.8	8.1	8.5	17.3
Digestive diseases	31.9	32.8	33.1	33.4	34.0	34.6	8.2
Cirrhosis and other chronic liver diseases	22.4	23.1	23.4	23.7	24.0	24.4	9.2
Neurological disorders	46.5	46.8	47.0	47.0	46.9	46.8	0.6
Mental disorders	14.5	14.5	14.5	14.5	14.5	14.5	0.1
Substance use disorders	3.5	3.5	3.4	3.4	3.4	3.3	-5.1
Diabetes and kidney disease:	11.8	12.1	12.5	12.8	13.4	14.1	20.0
-Diabetes mellitus	2.9	2.9	3.0	3.1	3.3	3.6	24.4
-Chronic kidney disease	10.2	10.5	10.8	11.1	11.5	12.1	19.0
Skin and subcutaneous diseases	25.7	25.8	25.8	25.9	26.0	26.3	2.2
Sense organ diseases	31.9	32.8	33.6	34.5	35.8	37.4	17.2
Musculoskeletal disorders	14.4	15.0	15.5	16.0	16.7	17.5	21.2
Other non-communicable diseases	65.2	65.2	65.5	65.7	65.9	66.1	1.3

NCD – non-communicable disease

*See **Table 1**.

For the population not living with HIV (ages 15+), the prevalence of neoplasms, cardiovascular diseases, and diabetes mellitus increases by about one-quarter between 2015 and 2040 (**Table 2**), a considerable increase but much slower than for PLWH. In comparison, because PLWH are, on average, about 5 years older than other adults, the prevalence of NCDs among PLWH is already higher than for the HIV-negative population as of 2017. This gap – in line with the growing age difference, and as PLWH increasingly reach older age brackets in which the prevalence of NCDs is considerably higher – will widen over the coming years. By 2040, the prevalence of neoplasms/cancers, cardiovascular diseases, and diabetes among PLWH will be more than double the prevalence for the respective diseases for adults not living with HIV.

Meanwhile, HIV prevalence is projected to decline over the projection period, eg, from 23.4% in 2015 to 12.4% for the adult population (ages 15+) by 2040 (see **Figure 2****,** panel B and **Table 3**). That is, while treatment and care for PLWH becomes more complex, the weight of this population declines. The pattern of HIV as a comorbidity of various NCDs or as a co-existing yet unrelated disease is uneven across disease categories (**Table 3**). For some diseases, the role of HIV as a comorbidity evolves similarly to adult HIV prevalence overall. However, for numerous categories of disease, the role of HIV as comorbidity or co-existing disease increases (cardiovascular diseases), changes barely (neoplasms/cancer), or declines very little (diabetes, respiratory diseases). This means that, for these important NCDs (the role of which increases because of population aging), the role of HIV as a comorbidity or co-existing disease does not diminish even though HIV prevalence declines.

**Table 3:** Prevalence of HIV among people affected by various NCDs, 2015-2040*

	HIV prevalence among population indicated (%)						
	**2015**	**2020**	**2025**	**2030**	**2035**	**2040**	**Relative increase, 2015-2040 (%)**
Populations affected by:							
Neoplasms/Cancers	25.9	28.6	29.6	29.5	28.0	25.3	-2.2
Cardiovascular diseases:	23.1	25.2	25.8	25.8	25.3	24.1	4.2
-Ischemic heart disease	23.9	26.7	28.1	28.5	28.1	26.9	12.6
-Cerebrovascular disease	24.0	26.8	28.1	28.5	27.9	26.3	9.8
-Hypertensive heart disease	21.5	24.0	25.3	26.1	26.7	27.0	25.3
-Cardiomyopathy and myocarditis	21.1	25.1	27.9	29.7	30.3	29.7	41.1
Chronic respiratory diseases	20.7	22.0	22.0	21.6	20.5	18.8	-9.4
Digestive diseases:	23.6	23.3	21.1	18.7	16.3	14.0	-40.9
-Cirrhosis and other chronic liver diseases	22.7	22.5	20.5	18.3	16.1	13.8	-39.2
Neurological disorders	21.3	20.3	17.6	15.0	12.6	10.4	-51.4
Mental disorders	20.2	19.6	17.3	15.1	13.0	11.0	-45.5
Substance use disorders	23.2	21.8	18.3	14.9	11.9	9.4	-59.5
Diabetes and kidney disease:	26.1	27.2	26.4	25.1	23.2	20.9	-19.9
-Diabetes mellitus	25.1	27.2	27.4	26.7	25.1	22.6	-10.0
-Chronic kidney disease	26.3	27.1	26.0	24.6	22.8	20.7	-21.5
Skin and subcutaneous diseases	15.3	15.3	14.3	13.4	12.3	11.2	-26.8
Sense organ diseases	25.9	26.3	25.0	23.1	20.8	18.2	-29.5
Musculoskeletal disorders:	27.2	27.9	26.5	24.6	22.2	19.5	-28.2
-Other non-communicable diseases	18.1	17.7	15.8	13.9	12.0	10.1	-44.1
Memorandum items:							
-HIV prevalence (total population)	16.6	16.2	14.4	12.7	11.0	9.4	-43.6
-HIV prevalence (ages 15+)	23.4	22.7	20.0	17.2	14.7	12.4	-47.0
-HIV prevalence (ages 60+)	17.4	22.0	26.3	30.8	34.3	34.7	99.2

NCD – non-communicable disease

*See **Table 1**.

The estimates reflect steep declines in HIV incidence envisaged under the draft NSF. In terms of the robustness of our findings, and for understanding the impacts of the policies envisaged, it is useful to understand the implications of this decline in incidence for the intersection of HIV and NCDs. If HIV incidence were to remain at estimated 2017 levels (at 1.3% annually), then HIV prevalence would increase from 16.6% in 2015 to 19.1% in 2040 rather than declining to 9.4% as in our projections (Table S4 in **Online Supplementary Document**). For ages 60+, the difference in outcomes on HIV prevalence is absolutely and relatively smaller – eg, prevalence rises to 41.3% rather than 34.7% by 2040. This reflects that most people who become infected with HIV in the period to 2040 do not reach old age within this period. Consequently, the HIV population increases faster but ages more slowly if the targets on reducing HIV incidence are not achieved. As a consequence, the prevalence of NCDs among PLWH is lower in this scenario (compared to the one with declines in incidence targeted under the NSF), while the projected prevalence of HIV among people affected by various NCDs comes out higher (see Tables S4 and S5 in **Online Supplementary Document**).

The estimates and projections so far did not take into account any impacts of HIV or ART on the prevalence of NCDs – reflecting the objective to isolate the effects of the aging of the population overall or living with HIV, the fact that the evidence is weak for most NCDs, and the uncertainties in projecting such linkages, eg, as drugs are adapted to mitigate any HIV-ART-NCD interactions. However, **Table 4** summarizes estimates on comorbidities between HIV on one hand, and ischemic heart disease and diabetes mellitus on the other, assuming – consistent with available empirical evidence – that the presence of HIV or a history of ART has increased the prevalence of the former by a factor of 1.5, and of the latter by a factor of 1.6 (see Appendix S3 in the in **Online Supplementary Document** on methods and further discussion on evidence) [34-42]. In this case, the prevalence of these NCDs among PLWH comes out higher, and HIV prevalence among populations affected by these diseases would reach close to 40%.

**Table 4:** Accounting for direct effects of HIV/AIDS on selected NCDs*

	Prevalence, for population indicated (%)						
	**2015**	**2020**	**2025**	**2030**	**2035**	**2040**	**Relative increase, 2015-2040 (%)**
Prevalence of selected NCDs among people living with HIV:							
-Ischemic heart disease	1.7	2.2	2.9	3.7	4.6	5.7	228.5
-Diabetes mellitus	4.3	5.2	6.4	7.7	9.2	10.6	143.5
HIV prevalence among population affected by selected NCDs:							
-Ischemic heart disease	31.8	35.0	36.2	36.3	35.4	33.4	5.0
-Diabetes mellitus	34.2	36.5	36.8	35.9	33.9	30.8	-9.9

NCD – non-communicable disease

*Assumes that, among people living with HIV, sex- and age-specific prevalence of cardiovascular disease is 50% higher, and prevalence of diabetes mellitus is 60% higher, compared to people not living with HIV.

## DISCUSSION

Some of our results mirror findings from the literature on the “greying of AIDS” and on the increasing role of NCDs among PLWH. As the population living with HIV ages, the prevalence of those NCDs which are more common at older ages increases among PLWH, and the health needs of PLWH become more complex in the presence of these co-morbidities [5-7,11].

The comprehensive demographic and population-level approach adopted here – focusing not only on PLWH but covering both HIV-positive and -negative people, generates important new insights for policy and practice. From this population perspective, the increase in the burden on health systems associated with the aging of the population living with HIV is not clear-cut – the health needs of PLWH become more complex, but HIV prevalence declines.

An additional factor that is often overlooked is past HIV-related mortality and its demographic implications. Cohorts reaching old age now have been decimated by AIDS-related mortality in middle age as many members of these cohorts contracted HIV and developed AIDS before ART was widely available. For this reason, HIV has been and is slowing down population aging, and it has reduced the share of the population at old age and the prevalence of age-related NCDs. However, as cohorts who have benefitted from the comprehensive treatment scale-up early on reach old age, this effect will dissipate, and the growth of the population at old age and of age-related NCDs will accelerate, – in Botswana, most notably between 2030 and 2040 (**Figure 1**, panel A).

In addition to the growing health needs of the population living with HIV, our findings introduce a second dimension through which HIV contributes to emerging health systems challenges. As observed elsewhere, population aging – irrespective of the impacts of HIV – will result in increasing prevalence of most NCDs in Botswana. We demonstrate that, even though HIV prevalence declines overall, the prevalence of HIV among people affected by many of the most important NCDs will increase or barely change. Because of the changing age profile of PLWH, HIV will thus be concentrated and persist in the growing populations facing the most complex health needs.

Some of our methods and results reflect the fact that estimated and projected treatment coverage in Botswana is already high, especially in older age cohorts where NCDs become more common. In countries where the scaling-up of treatment occurs later or is less complete, the projected acceleration in the growth of the older population would be delayed or less pronounced, and transitional factors associated with the scaling-up of treatment (recovery in BMI, reduced prevalence of AIDS-defining cancers) might have to be considered. However, the situation in Botswana resembles that in many other Southern African countries, in terms of very high HIV prevalence, a relatively high life expectancy before the onset of the HIV epidemic, and high ART coverage. More generally, a confluence of a high presence of HIV and an increasing burden of NCDs, coupled with a health system that is already stretched, is observed in many African countries, suggesting that our findings are relevant more widely [13].

Apart from the question as to what extent the results generalize, the principal limitation of the analysis arises from the simple assumptions on age-specific prevalence of NCDs and on HIV-NCD linkages. In this regard, it is encouraging that the results are robust to the two alternative assumptions on the trajectory of age-specific prevalence of NCDs. One item that will benefit from further work (but has not been captured for lack of solid evidence) is the interaction between HIV care and treatment and the onset of NCDs. To the extent that choices in HIV care and treatment affect the propensity to develop NCDs, our analysis may not capture fully the policy space for managing and shaping the joint HIV-NCD burden.

## CONCLUSIONS

In summary, health systems challenges posed by HIV/AIDS and its intersections with NCDs will grow and persist. The demographic impacts of HIV slow down overall population aging and thus the growing burden of many NCDs at present, but the lagged impacts of large-scale HIV treatment will accelerate population aging and increase chronic disease burden from about 2030. While HIV prevalence declines, the needs of PLWH become more complex. And HIV prevalence among the growing populations affected by various important NCDs will not decline for decades, leading to the persistent health systems challenge of how to successfully treat and care for the large numbers of patients suffering from both HIV and NCDs.

## Additional material

Online Supplementary Document
