# Supplementary material for: HIV and the growing health burden from noncommunicable diseases in Botswana: modelling study
Source: J Glob Health. 2019 May 12;9(1):010428. doi: 10.7189/jogh.09.010428 (PMC6607958; doi:10.7189/jogh.09.010428)
Supplement: Online Supplementary Document [file jogh-09-010428-s001.pdf]

J Glob Health 2019; 9: 010428

Supplementary Annex

Appendix S1.Alternative Estimates for Prevalence of Diabetes Mellitus

Annex Figure 1 summarizes available age- and sex-specific estimates on the prevalence of diabetes mellitus in Botswana, from the GBD 2017 database, the 2014 STEPS survey, and the 2017 IDF Atlas. We have decided to adopt the prevalence estimates from the 2014 STEPS survey, because they are directly based on national data. These data, however, are available for 4 age brackets only (15-29, 30-44, 44-59, and 60-69). For our analysis, we intrapolate the prevalence from the STEPS survey, as shown in Annex Figure 1, and extrapolate the estimate for the 65-69 age bracket for ages 70+.

Annex Figure 1. Alternative Estimates of the Prevalence of Diabetes Mellitus

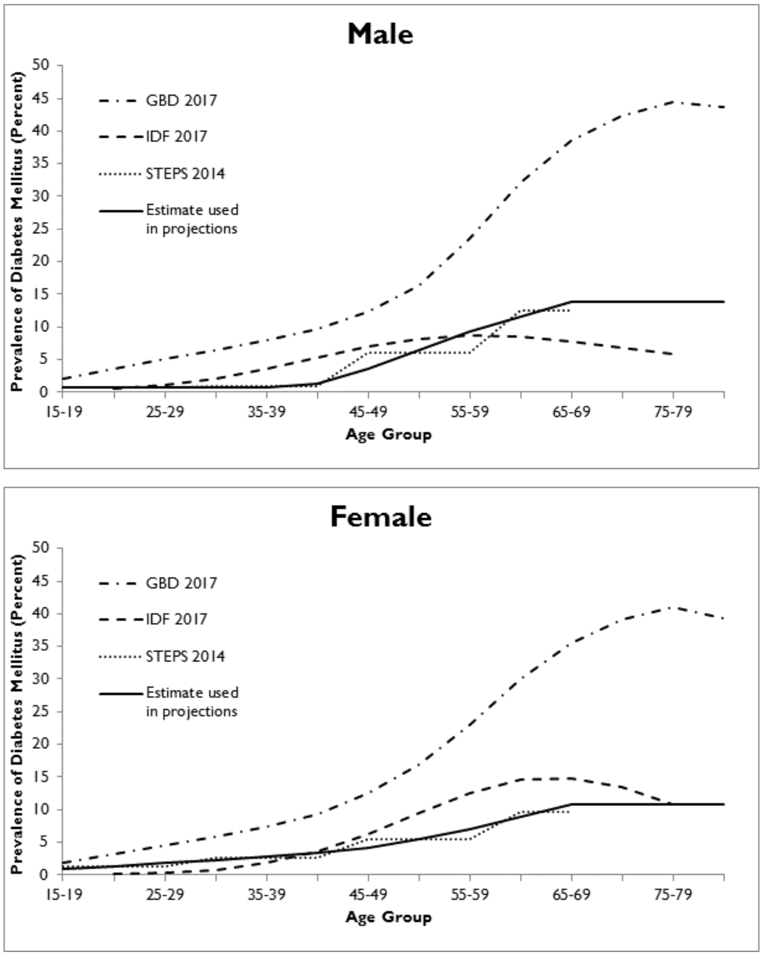

Because diabetes is a major risk factor for kidney disease (a factor taken into consideration in GBD estimates), adjusting estimates for diabetes has implications for prevalence of kidney disease. We have calculated prevalence of kidney disease among people suffering from diabetes and among those who are not from GBD estimates, and used these estimates to adjust estimates of the sex- and age-specific prevalence of kidney disease for lower prevalence of diabetes. Correspondingly, the adjusted sex- and age-specific prevalence of the combined GBD level 2 category "diabetes and kidney diseases" has been calculated as sex- and age-specific prevalence of diabetes, plus sex- and age-specific prevalence of chronic kidney disease among people not affected by diabetes, multiplied by the share of relevant share of the population not affected by diabetes.

Annex Figure 2. Alternative Estimates of the Prevalence of Chronic Kidney Disease

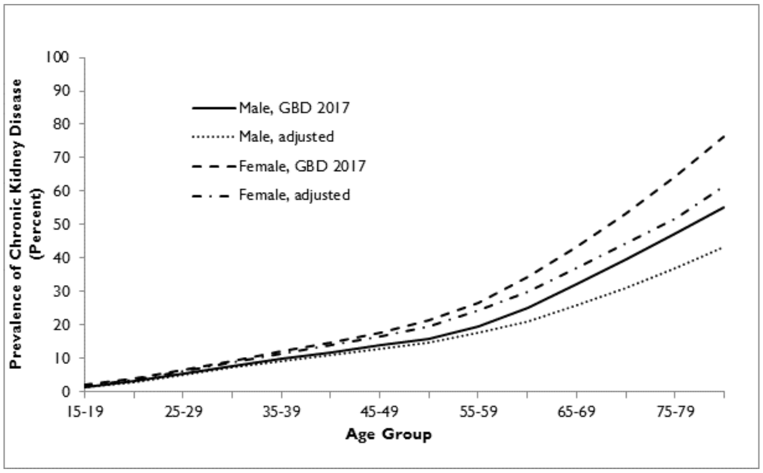

Because estimated prevalence of diabetes mellitus in the 2014 STEPS survey is less than one-half of prevalence estimates in the GBD 2017 database, our estimates of prevalence of diabetes among PLWH are accordingly lower than alternative estimates based on GBD 2017 data. Because the age profiles (adjusting for the overall level) are similar, though, the results on the prevalence of HIV among people affected by diabetes are robust. Estimated prevalence of diabetes (overall, and the increase by age) according to the IDF 2017 Atlas are similar to the data from the 2014 STEPS survey for the population overall, although there are differences by sex – IDF 2017 reports a lower build-up in prevalence by age for men, and a stronger one for women, compared to the results from the 2014 STEPS survey.

II. Appendix S2. Separate Projections for Men and Women

While we report estimates and projections for the population overall in the paper, the underlying analysis was done for men and women separately, in light of the straightforward availability of the relevant data differentiated by sex. Overall, the results come out similarly for the male and female populations, which is one reason why the paper focuses on the population overall. As an example for the separate results for men and women, Annex Tables 1A and 1B show estimates of the prevalence of NCDs among people living with HIV differentiated by sex.

Annex Table 1A. Prevalence of Selected NCDs Among Male Population Living with HIV, 2015-2040

	Prevalence (Percent)						Relative
	2015	2020	2025	2030	2035	2040	Increase, 2015-2040 (Percent)
Neoplasms/Cancers	0.3	0.4	0.6	0.8	1.1	1.4	344.7
Cardiovascular diseases	6.2	7.6	9.5	11.7	14.3	17.1	177.6
Ischemic heart disease	2.0	2.5	3.2	4.0	4.9	5.9	195.7
Cerebrovascular disease	0.9	1.2	1.5	1.9	2.4	2.8	208.8
Hypertensive heart disease	0.1	0.1	0.1	0.1	0.2	0.2	266.8
Cardiomyopathy and myocarditis	0.1	0.2	0.3	0.4	0.5	0.6	334.7
Chronic respiratory diseases	7.8	8.9	10.5	12.3	14.1	16.1	106.9
Digestive diseases	39.5	41.7	43.5	45.1	46.5	47.8	20.9
Cirrhosis and other chronic liver diseases	30.3	32.4	34.1	35.5	36.9	38.1	25.7
Neurological disorders	44.6	44.3	43.5	42.3	41.1	40.1	-10.0
Mental disorders	14.5	14.5	14.5	14.4	14.2	14.1	-3.0
Substance use disorders	4.0	3.9	3.7	3.5	3.4	3.2	-19.7
Diabetes and kidney disease	12.9	14.7	17.1	19.5	22.0	24.4	88.6
Diabetes mellitus	3.1	3.8	5.0	6.2	7.4	8.6	180.6
Chronic kidney disease	10.8	12.1	13.8	15.6	17.5	19.4	79.9
Skin and subcutaneous diseases	24.5	25.4	26.8	28.7	30.8	33.3	36.4
Sense organ diseases	38.9	42.8	48.0	53.0	57.6	61.9	59.2
Musculoskeletal disorders	19.5	21.9	24.7	27.3	29.8	32.0	63.8
Other non-communicable diseases	56.3	56.7	57.5	58.4	59.4	60.4	7.4

Notes: Table shows all "level 2" NCD categories from GBD 2017, and a selection of more specific diseases (indented). The relative increase is calculated as the ratio of prevalence in 2040 and 2015, respectively, minus one, multiplied by 100.

Annex Table 1A. Prevalence of Selected NCDs Among Male Population Living with HIV, 2015-2040

	Prevalence (Percent)						Relative
	2015	2020	2025	2030	2035	2040	Increase, 2015-2040 (Percent)
Neoplasms/Cancers	0.3	0.4	0.6	0.8	1.1	1.4	344.7
Cardiovascular diseases	6.2	7.6	9.5	11.7	14.3	17.1	177.6
Ischemic heart disease	2.0	2.5	3.2	4.0	4.9	5.9	195.7
Cerebrovascular disease	0.9	1.2	1.5	1.9	2.4	2.8	208.8
Hypertensive heart disease	0.1	0.1	0.1	0.1	0.2	0.2	266.8
Cardiomyopathy and myocarditis	0.1	0.2	0.3	0.4	0.5	0.6	334.7
Chronic respiratory diseases	7.8	8.9	10.5	12.3	14.1	16.1	106.9
Digestive diseases	39.5	41.7	43.5	45.1	46.5	47.8	20.9
Cirrhosis and other chronic liver diseases	30.3	32.4	34.1	35.5	36.9	38.1	25.7
Neurological disorders	44.6	44.3	43.5	42.3	41.1	40.1	-10.0
Mental disorders	14.5	14.5	14.5	14.4	14.2	14.1	-3.0
Substance use disorders	4.0	3.9	3.7	3.5	3.4	3.2	-19.7
Diabetes and kidney disease	12.9	14.7	17.1	19.5	22.0	24.4	88.6
Diabetes mellitus	3.1	3.8	5.0	6.2	7.4	8.6	180.6
Chronic kidney disease	10.8	12.1	13.8	15.6	17.5	19.4	79.9
Skin and subcutaneous diseases	24.5	25.4	26.8	28.7	30.8	33.3	36.4
Sense organ diseases	38.9	42.8	48.0	53.0	57.6	61.9	59.2
Musculoskeletal disorders	19.5	21.9	24.7	27.3	29.8	32.0	63.8
Other non-communicable diseases	56.3	56.7	57.5	58.4	59.4	60.4	7.4

Notes: Table shows all "level 2" NCD categories from GBD 2017, and a selection of more specific diseases (indented). The relative increase is calculated as the ratio of prevalence in 2040 and 2015, respectively, minus one, multiplied by 100.

Appendix S3 Alternative Projections of HIV-NCD Intersection

The results presented in the paper (Tables 1 to 4) are based on the assumption that the age-specific prevalence of NCDs will remain constant from 2017 – a crude assumption which is not implausible as estimated prevalence rates of NCDs (unlike mortality) in the Global Burden of Disease database change only slowly over time. One advantage of this approach is that the results can be attributed clearly to changes in the age structure of the relevant populations.

It would be similarly plausible to extrapolate based on trends in the prevalence of NCDs (although it is important to bear in mind that the scaling-up of antiretroviral therapy occurred from 2004, and the estimates would reflect any direct effects on this on the prevalence of NCDs). For this reason, and as a robustness check of the results reported in the paper, we reproduce key results (Tables 1 and 3) based on extrapolation of trends in 2010-2017 (Annex Tables 2 and 3). The sensitivity check excludes diabetes and kidney diseases, because we our estimates of the prevalence of diabetes is based on cross-sectional data from the 2014 STEPS survey rather than the GBD 2017 estimates of prevalence of NCDs over time.

Annex Table 2. Prevalence of Selected NCDS Among People Living with HIV, 2015-2040 If Sex- and Age-Specific Prevalence of NCDs Continues to Follow Trend in 2010-2017

	Prevalence (Percent)						Relative Increase, 2015-2040 (Percent)
	2015	2020	2025	2030	2035	2040	
Neoplasms/Cancers	0.6	0.7	0.9	1.1	1.4	1.7	197.4
Cardiovascular diseases	5.5	6.8	8.6	10.7	13.3	16.2	194.7
Ischemic heart disease	1.3	1.7	2.2	2.8	3.5	4.2	216.9
Cerebrovascular disease	0.9	1.2	1.6	2.2	2.9	3.7	321.9
Hypertensive heart disease	0.1	0.1	0.1	0.1	0.2	0.2	285.4
Cardiomyopathy and myocarditis	0.1	0.2	0.2	0.3	0.4	0.5	383.4
Chronic respiratory diseases	7.9	9.0	10.5	12.0	13.5	15.1	91.6
Digestive diseases	34.6	36.8	39.1	41.1	42.9	44.5	28.6
Cirrhosis and other chronic liver diseases	23.6	25.7	27.8	29.8	31.6	33.4	41.0
Neurological disorders	48.8	48.6	48.0	46.8	45.4	44.2	-9.4
Mental disorders	14.4	14.5	14.5	14.5	14.5	14.4	-0.1
Substance use disorders	3.2	3.1	2.9	2.6	2.4	2.2	-30.4
Diabetes and kidney disease	n.a.	n.a.	n.a.	n.a.	n.a.	n.a.	n.a.
Diabetes mellitus	n.a.	n.a.	n.a.	n.a.	n.a.	n.a.	n.a.
Chronic kidney disease	n.a.	n.a.	n.a.	n.a.	n.a.	n.a.	n.a.
Skin and subcutaneous diseases	24.7	25.5	26.9	28.8	31.1	33.9	37.4
Sense organ diseases	38.3	41.9	46.6	51.0	55.1	59.0	54.1
Musculoskeletal disorders	17.6	19.9	22.9	25.8	28.7	31.4	78.9
Other non-communicable diseases	69.0	69.1	69.1	68.7	68.2	67.7	-1.9

Based on extrapolation of trends in prevalence of NCDs, rather than assuming that age- and sex-specific prevalence remains constant from 2015.

Annex Table 3. Prevalence of HIV Among People Affected by Various NCDs, 2015-2040 If Sex- and Age-Specific Prevalence of NCDs Continues to Follow Trend in 2010-2017

	HIV prevalence among population indicated (Percent)						Relative
	2015	2020	2025	2030	2035	2040	Increase, 2015-2040 (Percent)
Populations affected by							
Neoplasms/Cancers	25.9	28.6	29.7	29.6	28.3	25.7	-0.8
Cardiovascular diseases	23.1	25.2	25.8	25.9	25.3	24.1	4.3
Ischemic heart disease	23.9	26.7	28.0	28.4	28.0	26.8	12.1
Cerebrovascular disease	24.0	26.7	28.0	28.3	27.6	26.1	8.9
Hypertensive heart disease	21.5	24.0	25.2	25.9	26.4	26.8	24.7
Cardiomyopathy and myocarditis	21.1	25.1	27.9	29.7	30.4	29.7	41.2
Chronic respiratory diseases	20.7	21.8	21.5	20.7	19.3	17.5	-15.4
Digestive diseases	23.6	23.3	21.2	18.8	16.4	14.0	-40.5
Cirrhosis and other chronic liver diseases	22.7	22.6	20.7	18.6	16.3	14.1	-38.1
Neurological disorders	21.3	20.3	17.6	15.0	12.6	10.4	-51.4
Mental disorders	20.2	19.6	17.3	15.1	13.0	11.0	-45.5
Substance use disorders	23.2	21.8	18.2	14.7	11.6	9.1	-61.0
Diabetes and kidney disease	n.a.	n.a.	n.a.	n.a.	n.a.	n.a.	n.a.
Diabetes mellitus	n.a.	n.a.	n.a.	n.a.	n.a.	n.a.	n.a.
Chronic kidney disease	n.a.	n.a.	n.a.	n.a.	n.a.	n.a.	n.a.
Skin and subcutaneous diseases	15.3	15.4	14.5	13.6	12.6	11.5	-24.9
Sense organ diseases	25.9	26.3	25.0	23.1	20.8	18.3	-29.3
Musculoskeletal disorders	27.2	27.9	26.6	24.7	22.3	19.7	-27.7
Other non-communicable diseases	18.1	17.7	15.9	14.0	12.0	10.2	-43.9
Memorandum items							
HIV prevalence (total population)	16.6	16.2	14.4	12.7	11.0	9.4	-43.6
HIV prevalence (ages 15+)	23.4	22.7	20.0	17.2	14.7	12.4	-47.0
HIV prevalence (ages 60+)	17.4	22.0	26.3	30.8	34.3	34.7	99.2

Based on extrapolation of trends in prevalence of NCDs, rather than assuming that age- and sex-specific prevalence remains constant from 2015.

Appendix S4. Alternative Estimates for Constant HIV Incidence

As a robustness check of our results with regard to the national HIV policies implemented or their effectiveness, we consider an alternative scenario in which HIV incidence remains constant from 2017 (at 1.3. percent annually). Results are summarized for prevalence of NCDs among people living with HIV (Annex Table 4, corresponding to Table 1), and HIV prevalence among people affected by various NCDs (Annex Table 5, corresponding to Table 3).

We do not provide a sensitivity analysis with respect to the pace of scaling up treatment, because coverage is already high at the outset. This means that most people access treatment relatively early, before reaching a stage of the disease characterized by high mortality. In this context, the most important outcome of faster scaling up – for the purposes of our projections –

is reduced HIV incidence through viral suppression captured in the sensitivity analysis on incidence.

Annex Table 4. Prevalence of Selected NCDS Among People Living with HIV, 2015-2040, If HIV Incidence Remains Constant at 2017 Level

	Prevalence (Percent)						Relative
	2015	2020	2025	2030	2035	2040	Increase, 2015-2040 (Percent)
Neoplasms/Cancers	0.6	0.7	0.8	0.9	0.9	1.0	77.6
Cardiovascular diseases	5.5	6.6	7.6	8.7	9.7	10.8	95.6
Ischemic heart disease	1.3	1.6	2.0	2.3	2.6	2.9	116.7
Cerebrovascular disease	0.9	1.1	1.3	1.5	1.8	2.0	123.7
Hypertensive heart disease	0.1	0.1	0.1	0.1	0.1	0.1	147.9
Cardiomyopathy and myocarditis	0.1	0.1	0.2	0.2	0.3	0.3	187.2
Chronic respiratory diseases	7.9	8.9	9.9	10.8	11.7	12.4	56.5
Digestive diseases	34.6	36.1	37.0	37.7	38.2	38.7	11.6
Cirrhosis and other chronic liver diseases	23.6	24.9	25.6	26.2	26.6	26.9	13.8
Neurological disorders	48.8	48.6	48.1	47.5	46.8	46.4	-4.8
Mental disorders	14.4	14.5	14.5	14.5	14.5	14.5	0.8
Substance use disorders	3.2	3.1	3.0	2.9	2.9	2.8	-12.0
Diabetes and kidney disease	13.7	15.4	17.0	18.5	19.8	21.0	53.2
Diabetes mellitus	3.2	3.7	4.3	4.7	5.2	5.5	71.5
Chronic kidney disease	11.9	13.3	14.5	15.7	16.9	17.9	50.4
Skin and subcutaneous diseases	24.7	25.4	26.2	27.2	28.1	29.1	18.0
Sense organ diseases	38.3	41.5	44.5	47.0	49.1	50.8	32.6
Musculoskeletal disorders	17.6	19.4	21.0	22.4	23.4	24.3	38.5
Other non-communicable diseases	69.0	69.3	69.5	69.5	69.4	69.5	0.6

Notes: Table shows all "level 2" NCD categories from GBD 2017, and a selection of more specific diseases (indented). The relative increase is calculated as the ratio of prevalence in 2040 and 2015, respectively, minus one, multiplied by 100.

Annex Table 5. Prevalence of HIV Among People Affected by Various NCDs, 2015-2040, If HIV Incidence Remains Constant at 2017 Level

	HIV prevalence among population indicated (Percent)						Relative Increase, 2015-2040 (Percent)
	2015	2020	2025	2030	2035	2040	
Populations affected by							
Neoplasms/Cancers	25.9	29.2	32.1	34.3	35.6	36.1	39.3
Cardiovascular diseases	23.1	25.9	28.5	30.7	32.7	34.1	47.6
Ischemic heart disease	23.9	27.3	30.3	32.9	35.0	36.5	52.8
Cerebrovascular disease	24.0	27.4	30.4	33.0	35.0	36.1	50.8
Hypertensive heart disease	21.5	24.5	27.4	30.1	32.8	35.5	65.1
Cardiomyopathy and myocarditis	21.1	25.5	29.7	33.2	36.0	37.9	80.1
Chronic respiratory diseases	20.7	22.7	24.8	26.6	28.0	28.9	39.5
Digestive diseases	23.6	24.4	25.1	25.7	26.2	26.5	12.3
Cirrhosis and other chronic liver diseases	22.7	23.6	24.4	25.0	25.6	26.0	14.4
Neurological disorders	21.3	21.4	21.6	21.8	22.2	22.4	5.2
Mental disorders	20.2	20.6	21.0	21.5	22.0	22.3	10.5
Substance use disorders	23.2	23.2	23.4	23.3	23.2	23.2	0.0
Diabetes and kidney disease	26.1	28.1	29.8	31.2	32.2	32.8	25.5
Diabetes mellitus	25.1	28.0	30.3	32.1	33.3	33.8	34.4
Chronic kidney disease	26.3	28.0	29.5	30.8	31.9	32.6	24.0
Skin and subcutaneous diseases	15.3	16.1	17.1	18.2	19.2	20.0	30.9
Sense organ diseases	25.9	27.3	28.6	29.6	30.3	30.7	18.5
Musculoskeletal disorders	27.2	28.8	30.1	31.1	31.7	32.1	17.9
Other non-communicable diseases	18.1	18.7	19.2	19.8	20.3	20.7	14.0
Memorandum items							
HIV prevalence (total population)	16.6	17.1	17.6	18.2	18.8	19.1	15.4
HIV prevalence (ages 15+)	23.4	24.0	24.4	24.6	24.9	25.3	7.9
HIV prevalence (ages 60+)	17.4	22.4	27.8	33.6	38.8	41.3	137.2

Notes: See Table 1.

Appendix S5Estimating and Projecting HIV-NCD Comorbidities

One important factor in our estimates is the form of the link between HIV status and the prevalence of NCDs. To the extent that these are positively correlated within the categories by sex and age we are using, for behavioural reasons (if an NCD risk factor is more common among PLWH than otherwise) or reflecting direct effects of HIV or long-term treatment on NCDs, our estimates of the prevalence of NCDs among PLWH and of HIV among people affected by NCDs would be underestimates.

There is a considerable literature on HIV-NCD Comorbidities, including a comprehensive survey of the state of knowledge in the special issue of the *Journal of Acquired Immune Deficiency Syndromes* on "HIV Noncommunicable Disease Comorbidities in Low- and Middle-Income Countries in the ART Era" of September 2014. However, evidence across NCDs on such

HIV-NCD linkages is uneven, and many studies do not provide estimates for matching populations not affected by HIV/AIDS, and the link between HIV and NCDs is likely endogenous, depending on types of drugs and factors like treatment initiation and duration (Althoff, Smit, Reiss, and Justice, 2016), and changing over time (Vachiat and others, 2017). Moreover, the bulk of the evidence is based on data from high-income countries, and their results may not translate well into the context of Southern Africa (Levitt, Steyn, Dave, and Bradshaw (2011), Mosepele and Botsile (2018)).

In light of these uncertainties, and considering the possibility that the link between HIV and NCDs may change over the next decades as drug regimens are optimized to reduce adverse consequences of long-term ART (plausible in light of the evidence on risk of cardiovascular disease and different types of antiretroviral drugs used so far, see Islam, Wu, Jansson, and Wilson (2012); Martin-Iguacel, Llibre, and Friis-Moller (2015); Vachiat and others (2017)), we do not include an elevated risk of developing NCDs among PLWH in most of our results. However, we report alternative estimates for ischemic heart disease and diabetes mellitus (see Table 4 of main paper), two diseases for which the evidence on HIV-NCD links is relatively strong, and which are important contributors to the burden of disease in Botswana, ranking 2nd and 5th, respectively, among causes of death (with HIV still being the no. 1 cause).

For ischemic heart disease, we assume in the alternative specification that prevalence is elevated by a factor of 1.5 among people with HIV, controlling for sex and age. This factor is consistent with and at the lower end of results reported in comprehensive surveys specifically on HIV and ischemic heart disease (Vachiat and others (2017) or HIV and cardiovascular diseases more generally (Islam, Wu, Jansson, and Wilson (2012); Martin-Iguacel, Llibre, and Friis-Moller (2015), which report a relative risk of cardiovascular disease among PLWH (compared to HIV-negative people) of between 1.5 and 2.0.

For diabetes, sex- and age-specific prevalence among PLWH is elevated by a factor of 1.6, relative to HIV-negative people in that population group. This estimate follows Prioreschi and others (2017), who synthesize available evidence from sub-Saharan Africa (but point to a high variation among the estimates from the underlying studies).

While there is a considerable empirical literature on links between HIV/AIDS and cancers, we did not include cancers here because most of the literature regards the declining role of AIDS-defining cancers following introduction of antiretroviral therapy (which is not important here because treatment coverage is already high at the beginning of the projection) and the subsequent increase in the role of non-AIDS defining cancers, largely in line with the aging of the population living with HIV (Chinula,Moses, and Gopal, 2017), a factor we do capture. Moreover, the evidence is heterogeneous with regard to types of cancer and different types of antiretroviral therapy (Borges and others (2017)).

The sex-and age-specific prevalence of the selected NCDs (ischemic heart disease and diabetes mellitus), among PLWH and otherwise, has been estimated with the following equation:

$$\mathrm{Prev}_{\mathrm{NCD|Total}} = \mathrm{Prev}_{\mathrm{HIV|Total}} \cdot \mathrm{Prev}_{\mathrm{NCD|PLWH}} + \left(100 - \mathrm{Prev}_{\mathrm{HIV|Total}}\right)\frac{\mathrm{Prev}_{\mathrm{NCD|PLWH}}}{\mathrm{M}_{\mathrm{NCD}}}, \qquad \text{(A1)}$$

where $\text{Prev}_{X|Y}$ denotes the prevalence of disease X among the total population (Y=”Total”) or PLWH (Y=PLWH) in the respective (sex and age) bracket. M_{NCD} is the factor by which prevalence of an NCD is elevated among PLWH, relative to the population not living with HIV. Using available estimates of $\text{Prev}_{NCD|Total}$, $\text{Prev}_{HIV|Total}$, by sex and age bracket, and the assumed level of M_{NCD}, Eq. (1) can be solved for the prevalence of an NCD among PLWH ($\text{Prev}_{NCD|PLWH}$) and the prevalence of an NCD among people not living with HIV, $\frac{\text{Prev}_{NCD|PLWH}}{M_{NCD}}$.

The implied assumption that M_{NCD} is constant across sex and age groups may be restrictive. To introduce variations in M_{NCD} by sex or age, we would require corresponding empirical estimates, or need to adopt an explicit model of NCD incidence and progress with and without NCDs. Empirical studies differentiating M by sex or age are rare, one such study (Althoff and others (2015), focusing on incidence of myocardial infarction, renal disease, and non-AIDS-defining cancer) suggests that the factor M is even across ages. One study adopting an more explicit modeling approach (Smit and others (2018), on Zimbabwe) concludes that results are primarily driven by the changing age profile of people living with HIV rather than the cumulative exposure to HIV and ART, and points to the limitations imposed by sparse data availability for age-specific NCD incidence or prevalence estimates.

References

Althoff, Keri N., and others, 2015, “Comparison of Risk and Age at Diagnosis of Myocardial Infarction, End-Stage Renal Disease, and Non-AIDS-Defining Cancer in HIV-Infected Versus Uninfected Adults,” Clinical Infectious Diseases, Vol. 60, No. 4, pp.627-638.

Althoff, Keri N., Mikaela C. Smit, Peter C. Reiss, and Amy C. Justice, 2016, "HIV and Ageing: Improving Quantity and Quality of Life," Current Opinion in HIV and AIDS, Vol. 11, No. 5, p.527-536.

Borges, H., Álvaro, 2017, "Combination Antiretroviral Therapy and Cancer Risk," Current Opinion in HIV and AIDS, Vol.12, No. 1, pp.12-19.

Chinula, Lameck, Agnes Moses, and Satish Gopal, 2017, "HIV-Associated Malignancies in Sub-Saharan Africa: Progress, Challenges, and Opportunities," Current Opinion in HIV and AIDS, Vol. 12, No. 1, pp.89-95.

Freiberg, Matthew S., and others, 2013, "HIV Infection and the Risk of Acute Myocardial Infarction," JAMA Internal Medicine, pp.1-9, Vol. 173, No. 8, pp. 614-622.

Islam, F.M., J. Wu, J. Jansson, and D.P. Wilson, 2012, "Relative Risk of Cardiovascular Disease among People Living with HIV: A Systematic Review and Meta-Analysis," *HIV Medicine*, Vol. 13, No. 8, pp.453-468.

Institute for Health Metrics and Evaluation (IHME), 2018, Global Burden of Disease 2017 Database (Seattle: IHME. 2018).

International Diabetes Foundation. 2017, IDF Diabetes Atlas, Eighth Edition (Brussels: International Diabetes Foundation).

Levitt, Naomi S., Krisela Steyn, Joel Dave, and Debbie Bradshaw, 2011, "Chronic Noncommunicable Diseases and HIV-AIDS on a Collision Course: Relevance For Health Care Delivery, Particularly in Low-Resource Settings—Insights from South Africa," American Journal of Clinical Nutrition, Vol. 94 (Suppl.), pp. 1690S–6S.

Martin-Iguacel, R., J. M. Llibre, and N. Friis-Moller, 2015, "Risk of Cardiovascular Disease in an Aging HIV Population: Where Are We Now?" Current HIV/AIDS Reports, Vol. 12, No. 4, pp. 375–87.

Ministry of Health and World Health Organisation, 2015, Botswana STEPS Survey Report On Non-Communicable Disease Risk Factors (Gaborone: Ministry of Health).

Mosepele, Mosepele, and Elizabeth Botsile, 2018, "Role of Noncommunicable Diseases in Resource-Limited Settings," Current HIV/AIDS Reports, Vol.15, No. 2, pp.120-126.

Prioreschi, Alessandra, and others, 2017, "Incidence and Prevalence of Type 2 Diabetes Mellitus with HIV Infection in Africa: A Systematic Review and Meta-Analysis," BMJ Open, Vol.7, No. 3:e013953.

Smit, Mikaela, and others, 2015, "Future Challenges for Clinical Care of an Ageing Population Infected with HIV: A Modelling Study," The Lancet Infectious Diseases, Vol.15, No. 7, pp. 810-818.

Vachiat, Ahmed, and others, 2017, "HIV and Ischemic Heart Disease," Journal of the American College of Cardiology, Vol.69, No. 1, pp. 73-82.
